# Independent and combined predictive value of nutritional, electrolyte, inflammatory, and insulin resistance indices for post-stroke infection in acute ischemic stroke: a retrospective study

**DOI:** 10.3389/fnut.2026.1863416

**Published:** 2026-08-12

**Authors:** Jianmo Liu, Canwei Yi, Haowen Luo, Pengfei Yu, Zhaofa Liu, Hongfei Zhao, Huiming Liao, Xiao Zhang, Weijiang Han, Chumin Zhou, Yingping Yi

**Affiliations:** 1 Department of Medical Big Data Research Centre, The Second Affiliated Hospital of Nanchang University, Nanchang, China; 2 Jiangxi Provincial Key Laboratory of Preventive Medicine, School of Public Health, Nanchang University, Nanchang, China

**Keywords:** electrolyte index, inflammatory index, insulin resistance index, nutritional index, post-stroke infection, prediction model

## Abstract

**Background:**

Post-stroke infection (PSI) is a major cause of poor prognosis in acute ischemic stroke (AIS). The independent and combined predictive value of nutritional, electrolyte, inflammatory, and insulin resistance indices for PSI requires further validation.

**Methods:**

We retrospectively analyzed 6,149 AIS patients from the Second Affiliated Hospital of Nanchang University (June 2017–April 2025). Multivariate logistic regression, restricted cubic spline (RCS), and ROC curves assessed the predictive value of nutritional (GNRI, PNI), electrolyte (serum sodium, potassium), inflammatory (NLR, PLR), and insulin resistance (TyG, TyG-BMI) indices. Hierarchical clustering selected clinical features; five ML algorithms (XGBoost, RF, LightGBM, Extra Trees, CatBoost) with 5-fold cross-validation built predictive models. SHapley Additive exPlanations (SHAP) identified key predictors.

**Results:**

NLR, PLR and TyG were elevated in PSI patients, while GNRI, PNI, and serum sodium were decreased (all *p* < 0.05). RCS identified malnutrition (GNRI ≤98.799, PNI ≤ 46.898), a U-shaped sodium–PSI relationship (inflection: 139.611 mmol/L), elevated NLR (>2.703), and elevated TyG as risk factors; After adjusting for confounding factors such as comorbidities, the statistical significance of potassium metabolic disturbances disappeared (*p* = 0.151), suggesting that their impact on PSI may be due to confounding by underlying diseases rather than an independent causal association. The non-linear potassium–PSI association (inflection: 3.529 mmol/L) was not significant in the fully adjusted model. TyG-BMI was not independently associated with PSI. The CatBoost model integrating all four biomarker categories with clinical features performed best (AUC = 0.750, 95% CI: 0.722–0.779). SHAP identified TyG (|SHAP| = 0.409), GNRI (0.358), NLR (0.293), invasive procedure (0.194), coronary heart disease (0.179), and atrial fibrillation (0.137) as top features.

**Conclusion:**

Nutritional, electrolyte, inflammatory, and insulin resistance indices are significantly associated with PSI, and their integration with clinical features produces a predictive model with good discriminative performance.

## 1 Introduction

As one of the neurological diseases with the highest disability and mortality rates worldwide, the prognosis of stroke not only depends on the degree of neurological function damage but is also closely related to various systemic complications (1, 2). Among these, PSI is a leading contributor to adverse outcomes, significantly increases patient mortality, prolongs hospital stays, and delays neurological recovery, imposing a heavy burden on clinical treatment and public health systems (3, 4). Therefore, early identification of high-risk patients for PSI and implementation of targeted interventions are key strategies for improving patient outcomes.

In recent years, an increasing number of studies have demonstrated that nutritional status, electrolyte balance, inflammatory response, and metabolic disorders play significant roles in the development of PSI (5). Nutritional indices such as the Geriatric Nutritional Risk Index (GNRI) and Prognostic Nutritional Index (PNI) comprehensively reflect an individual’s immune-nutritional status by integrating indicators like serum albumin and lymphocyte count. Their reduction is closely associated with an increased risk of infection, and they have been proven to have prognostic assessment value in patients with stroke (6, 7). Electrolyte imbalance may further increase the risk of infection by affecting immune cell function, intestinal barrier integrity, and neuroendocrine regulation (8, 9). The inflammatory markers neutrophil-to-lymphocyte ratio (NLR) and platelet-to-lymphocyte ratio (PLR), as readily accessible systemic inflammation indicators, have been demonstrated to be significantly associated with prognosis and infection risk in stroke patients. Elevated levels reflect immune imbalance and exacerbated inflammatory states (10). Additionally, insulin resistance indices such as the triglyceride-glucose index (TyG) and triglyceride-glucose body mass index (TyG-BMI) may be associated with infection risk by assessing the severity of metabolic disorders, as they are closely linked to chronic inflammation, immunosuppression, and vascular endothelial dysfunction (11). Although the predictive value of the aforementioned indicators has been validated in single diseases such as tumors and cardiovascular diseases, research on combined multi-indicator assessment remains scarce in the field of PSI. Currently, most studies focus on single indicators (such as NLR or nutritional status), and no study has systematically constructed and validated a combined predictive model integrating nutritional, electrolyte, inflammatory, and insulin resistance indicators. Therefore, this study aims to systematically explore the correlations between PNI, GNRI, serum sodium,serum potassium, NLR, PLR, TyG, TyG-BMI and PSI, and to evaluate their combined predictive value, in order to provide a theoretical basis for early clinical identification of high-risk patients and the formulation of individualized prevention and treatment strategies.

## 2 Methods

### 2.1 Data sources and study population

**Figure 1:**
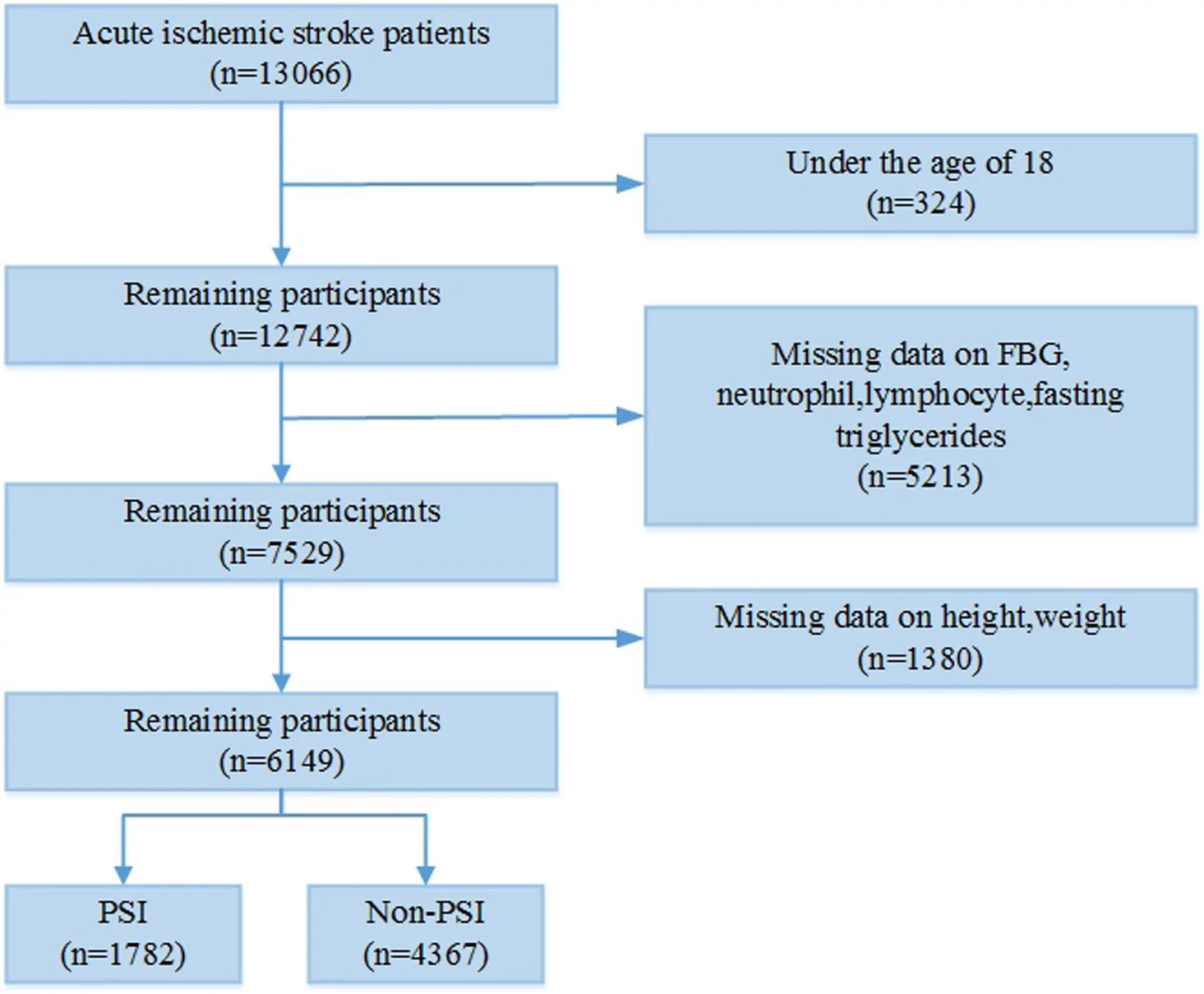
Flowchart of the patient selection process.

In this study, a cohort of 13,066 patients diagnosed with AIS was initially identified from the Second Affiliated Hospital of Nanchang University, spanning the period from June 2017 to April 2025. AIS was defined as an episode of neurological dysfunction caused by focal cerebral infarction confirmed by neuroimaging, according to the tissue-based criteria proposed by the AHA/ASA (12). Inclusion criteria required patients to be aged 18 years or older, admitted to the hospital within 48 h of AIS symptom onset. To ensure the integrity of the dataset, the following exclusions were applied: 324 patients under the age of 18; 5,213 patients with missing data on fasting blood glucose (FBG), neutrophil count, lymphocyte count, and fasting triglycerides index; and 1,380 patients with incomplete measurements of height and weight. Consequently, 6,149 patients were included in the final analysis. A comprehensive flowchart detailing participant inclusion is presented in Figure 1. PSI is defined as infections occurring within 7 days of stroke onset, excluding pre-existing or incubation-period infections, with respiratory and urinary tract infections being most common (13). All predictor variables—including laboratory test results and records of invasive procedures—were collected strictly within the first 48 h following hospital admission. The outcome variable, post-stroke infection (PSI), was defined as any newly diagnosed infection occurring beyond 48 h after admission. This temporal framework ensures a clear chronological sequence between exposure and outcome, thereby minimizing potential bias arising from infections present at the time of admission.

Among 6,149 patients with acute ischemic stroke, 1,782 developed PSI, yielding an infection rate of 28.98%. The distribution of infection types was as follows: pulmonary infection (*n* = 1,621), gastrointestinal infection (*n* = 49), urinary tract infection (*n* = 282), skin and soft tissue infection (*n* = 9), and mixed-site infection (*n* = 172), as shown in Table 1.

**Table 1:** Distribution of post-stroke infection sites among 1,782 stroke patients.

Infection site	*n*	%
Pulmonary infection	1,621	90.96
Gastrointestinal infection	49	2.75
Urinary tract infection	282	15.83
Skin and soft tissue infection	9	0.51
Mixed-site infection	172	9.65

### 2.2 Data collection

The following data was collected in this study:

- Demographics: sex, age;
- Life style: smoking status, alcohol consumption status;
- Body measurements: height, weight, pulse;
- Clinical presentation on admission: disorders of consciousness;
- In-hospital procedures: invasive procedure;
- Past medical history: diabetes, hypertension, atrial fibrillation, renal insufficiency, coronary heart disease, stroke;
- Laboratory tests: FBG, fasting triglycerides, total cholesterol, neutrophil count, lymphocyte count, platelet count, monocyte count, albumin, C-reactive protein(CRP),serum sodium, serum potassium, white blood cell count, creatinine, neutrophil ratio, total protein, Low density lipoprotein cholesterol(LDL-C), High density lipoprotein cholesterol(HDL-C) were performed within 24 h of admission.

### 2.3 Data assessment and definitions

BMI, GNRI, PNI, NLR, PLR, TyG, TyG-BMI are calculated via the following formulas:

$\mathrm{BMI}=\text{weight}\;(\mathrm{kg})/\text{height}\;{\left(m\right)}^{2}$ (14).

GNRI=1.489×albumin(g/L)+41.7×[weight/ideal weight] (15).

Where ideal weight = 22 × height (m) × height (m). If the actual weight exceeds the ideal weight, the weight/ideal weight ratio is set to 1.

$\mathrm{PNI}=\text{albumin}\;(g/L)+5\times \text{lymphocyte count}\;(\times 10^{9}/L)$ (16).

$\begin{array}{c} \mathrm{NLR}\;(\text{Neutrophil}-\text{to}-\text{Lymphocyte Ratio})=\text{Neutrophil count}\\ /\text{Lymphocyte count} \end{array}$ .

$\begin{array}{c} \mathrm{PLR}\;(\text{Platelet}-\text{to}-\text{Lymphocyte Ratio})=\text{Platelet count}\\ /\text{Lymphocyte count} \end{array}$ .

$\mathrm{TyG}=\ln\;(\begin{array}{l} \text{fasting triglyceride}\;(\mathrm{mg}/\mathrm{dL})\\ \times \text{fasting glucose}\;(\mathrm{mg}/\mathrm{dL}) \end{array})/2$ , since the concentrations of triglyceride and glucose in clinical tests are often measured in millimoles per liter (mmol/L), unit conversion is required: fasting triglyceride (mg/dL) = fasting triglyceride (mmol/L) × 88.57, fasting glucose (mg/dL) = fasting glucose (mmol/L) × 18.

$\mathrm{TyG}-\mathrm{BMI}=\mathrm{TyG}\times \mathrm{BMI}\;(\mathrm{kg}/m^{2})$ .

### 2.4 Statistical analysis

Statistical analyses were conducted utilizing SPSS version 22.0 and Python version 3.7. Continuous variables that conformed to a normal distribution were presented as mean ± standard deviation (SD), with intergroup comparisons executed via t-tests. Conversely, for continuous variables that did not conform to a normal distribution, data were expressed as median with interquartile range [M(IQR)], and intergroup comparisons were performed using non-parametric tests. Categorical data were described by frequency and percentage (%), with intergroup comparisons conducted using the chi-square test or Fisher’s exact test. A *p*-value of less than 0.05 was considered indicative of statistical significance. The study cohort was randomly partitioned into a training set (80%) and a test set (20%). During model development, we used five machine learning algorithms: XGBoost, Random Forest (RF), Light Gradient Boosting Machine (LightGBM), Extra Trees (ET), and Categorical Boosting (CatBoost). Each algorithm was trained and tuned via 5-fold cross-validation on the training set. We then compared their performance on a held-out test set and selected the model with the best predictive ability. The predictive performance of the models was assessed using metrics such as sensitivity, specificity, Youden Index, and the area under the receiver operating characteristic curve (AUC).

## 3 Results

### 3.1 Comparison of clinical characteristics between PSI and non-PSI groups in the training set

In the training set, a total of 4,919 patients were included, with a median (IQR) age of 67 (58–74) years. The cohort comprised 36.00% females and 64.00% males, and 1,444 individuals (29.36%) developed PSI. Compared with the non-PSI group, patients in the PSI group were significantly older and had higher pulse rates, as well as a greater prevalence of hypertension, diabetes, atrial fibrillation, coronary heart disease, renal insufficiency, disorders of consciousness, and invasive procedures. Furthermore, the PSI group exhibited significantly elevated levels of neutrophil count, white blood cell count, monocyte count, neutrophil ratio, FBG, creatinine, fasting triglycerides, CRP, NLR, PLR, serum potassium and TyG. In contrast, levels of lymphocyte count, platelet count, albumin, total protein, total cholesterol, LDL-C, BMI, GNRI, PNI and serum sodium were significantly lower in the PSI group (*p* < 0.05) (Table 2).

**Table 2:** Comparison of clinical characteristics between PSI and non-PSI groups in the training set.

Variable	Non-PSI (*n* = 3,475)	PSI (*n* = 1,444)	Statistic	*p*-value
Demographics				
Sex-male, (%)	2,244 (64.6%)	904 (62.6%)	1.637	0.2008
Age, years	65.00 (56.00–72.00)	72.00 (63.00–79.00)	−17.852	<0.001
Life style				
Smoking, (%)	738 (21.2%)	329 (22.8%)	1.347	0.2458
Drinking, (%)	661 (19.0%)	297 (20.6%)	1.458	0.2272
Body measurements				
Height, cm	165.00 (158.00–169.00)	165.00 (157.00–168.00)	2.326	0.0200
Weight, kg	64.00 (55.00–71.00)	62.00 (54.00–69.00)	6.674	<0.001
BMI	23.83 (21.83–25.88)	23.19 (21.26–25.10)	7.096	<0.001
Pulse, bpm	86.00 (82.00–90.00)	89.00 (84.00–96.00)	−11.843	<0.001
Past medical history				
Stroke, (%)	10 (0.3%)	2 (0.1%)	–	0.5276
Hypertension, (%)	2,611 (75.1%)	1,161 (80.4%)	15.521	<0.001
Diabetes, (%)	1,143 (32.9%)	551 (38.2%)	12.296	<0.001
Atrial fibrillation, (%)	167 (4.8%)	227 (15.7%)	163.448	<0.001
Coronary heart disease, (%)	374 (10.8%)	327 (22.6%)	116.903	<0.001
Renal insufficiency, (%)	190 (5.5%)	253 (17.5%)	179.377	<0.001
Clinical presentation on admission				
Disorders of consciousness,(%)	93 (2.7%)	232 (16.1%)	294.249	<0.001
In-hospital procedures				
Invasive procedure, (%)	64 (1.8%)	215 (14.9%)	322.151	<0.001
Laboratory tests				
Neutrophil count, ×10^9^/L	4.27 (3.33–5.59)	4.79 (3.56–6.78)	−8.895	<0.001
Lymphocyte count, ×10^9^/L	1.61 (1.23–2.03)	1.38 (1.01–1.81)	11.424	<0.001
Platelet count, ×10^9^/L	203.00 (168.00–245.00)	198.00 (160.00–239.00)	3.554	<0.001
White blood cell count, ×10^9^/L	6.68 (5.50–8.17)	7.01 (5.68–9.17)	−6.038	<0.001
Albumin, g/L	39.28 (36.83–41.86)	37.78 (34.94–40.60)	11.837	<0.001
Total protein, g/L	65.90 (62.29–70.27)	65.43 (61.09–69.70)	3.826	<0.001
CRP, mg/L	2.84 (1.56–6.08)	6.92 (2.26–33.97)	−17.644	<0.001
Monocyte count, ×10^9^/L	0.43 (0.33–0.56)	0.46 (0.34–0.64)	−5.175	<0.001
Neutrophil ratio, %	65.40 (58.70–72.65)	70.20 (61.30–79.10)	−11.822	<0.001
FBG, mmol/L	5.32 (4.71–6.56)	5.68 (4.80–7.43)	−6.232	<0.001
Creatinine, μmol/L	73.77 (61.97–88.32)	77.60 (64.53–96.50)	−6.318	<0.001
Total cholesterol, mmol/L	4.46 (3.73–5.20)	4.32 (3.59–5.09)	4.426	<0.001
Fasting triglycerides, mmol/L	1.13 (0.80–1.58)	1.35 (0.94–2.03)	−11.277	<0.001
LDL-C, mmol/L	2.46 (1.91–3.11)	2.29 (1.66–2.96)	6.297	<0.001
HDL-C, mmol/L	1.10 (0.93–1.34)	1.12 (0.91–1.34)	0.263	0.7929
Nutritional index				
GNRI	99.59 (95.47–103.57)	96.88 (92.28–101.42)	12.477	<0.001
PNI	47.63 (44.22–51.20)	45.15 (41.30–49.00)	14.484	<0.001
Electrolyte index				
Serum sodium, mmol/L	139.80 (137.91–141.55)	139.24 (137.05–141.30)	5.283	<0.001
Serum potassium, mmol/L	3.83 (3.57–4.07)	3.86 (3.60–4.17)	−4.096	<0.001
Inflammation index				
NLR	2.61 (1.87–3.79)	3.37 (2.20–5.95)	−12.895	<0.001
PLR	126.40 (97.69–166.67)	143.43 (103.55–197.53)	−8.572	<0.001
Insulin resistance index				
TyG	8.52 (8.14–8.98)	8.78 (8.33–9.29)	−11.127	<0.001
TyG-BMI	204.49 (182.98–226.37)	203.71 (183.22–225.08)	0.274	0.7838

### 3.2 Nutrition, electrolyte, inflammation, insulin resistance indices of the training set

**Figure 2:**
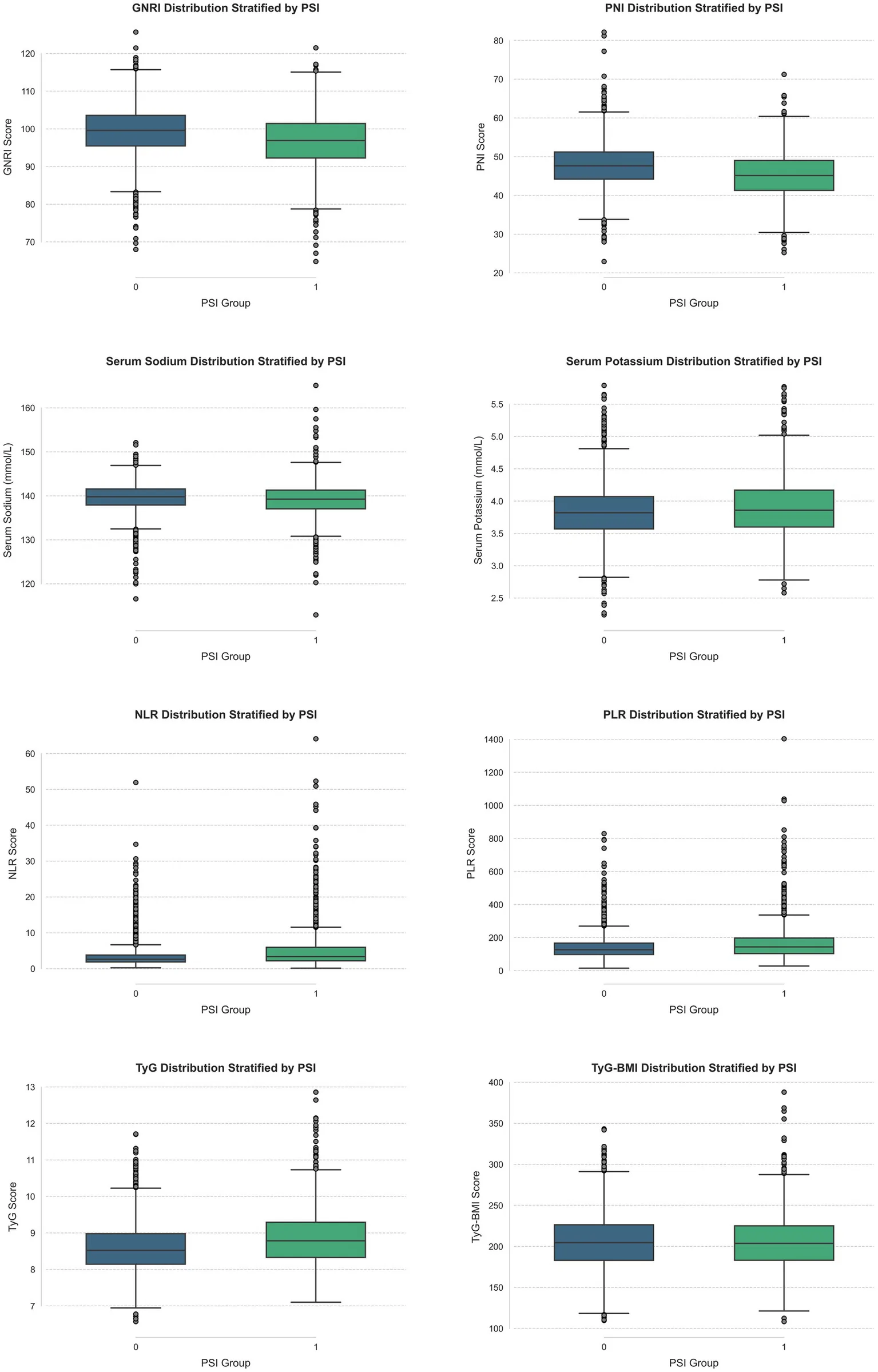
Violin plots of nutrition index, electrolyte index, inflammation index, insulin resistance index in the Non-PSI and PSI groups.

As shown in Table 2, nutrition, electrolyte, inflammation, and insulin resistance indices differed significantly between the two groups in the training set. Specifically, patients in the PSI group had significantly higher levels of NLR (3.37 [2.20–5.95] vs. 2.61 [1.87–3.79]), PLR (143.43 [103.55–197.53] vs. 126.40 [97.69–166.67]),and TyG (8.78 [8.33–9.29] vs. 8.52 [8.14–8.98]) than those in the non-PSI group. In contrast, nutrition-related indices, including GNRI (96.88 [92.28–101.42] vs. 99.59 [95.47–103.57]) and PNI (45.15 [41.30–49.00] vs. 47.63 [44.22–51.20]), as well as the serum sodium level (139.24 [137.05–141.30] vs. 139.80 [137.91–141.55]), were significantly lower in the PSI group (*p* < 0.001) (Figure 2).

### 3.3 Association analysis of nutrition, electrolyte, inflammation, insulin resistance indices with PSI

To clarify the association between nutritional, electrolyte, inflammatory, and insulin resistance indicators with PSI, this study developed three progressively adjusted logistic regression models: Model 1 (unadjusted), Model 2 (adjusted for age and gender), and Model 3 (further adjusted for past medical history). The findings indicate that both the GNRI and the PNI are significantly negatively associated with PSI risk across all models. This association remains consistent even after controlling for additional variables (Model 3: GNRI OR = 0.964, 95% CI = 0.954–0.974; PNI OR = 0.943, 95% CI = 0.931–0.955), suggesting that low nutritional status is a risk factor. A significant correlation was also identified between reduced serum sodium levels and an increased risk of PSI, with this relationship persisting after adjustments (Model 3: OR = 0.967, 95% CI = 0.948–0.986). In contrast, for serum potassium, an elevated risk was only apparent in the unadjusted model and when demographic characteristics were considered (Model 1: OR = 1.404; Model 2: OR = 1.272). However, this association disappeared upon the inclusion of past medical history (Model 3: OR = 1.122, *p* > 0.05). These findings suggest that the observed relationship between serum potassium and PSI risk may be confounded by underlying medical conditions. In all three models, the NLR and PLR demonstrated a significant positive correlation with the risk of PSI, with the adjusted effects remaining significant, albeit slightly diminished (Model 3: NLR OR = 1.108, 95% CI = 1.089–1.128; PLR OR = 1.003, 95% CI = 1.003–1.004). This indicates that an elevated systemic inflammatory response serves as a strong predictor. TyG index also exhibited a significant positive correlation with PSI risk, with the effect size increasing as additional adjustment variables were incorporated (Model 3: OR = 2.057, 95% CI = 1.848–2.293). Conversely, the TyG-BMI showed a significant association in Model 2 (OR = 1.004, *p* < 0.001), but this association was attenuated and became non-significant in the fully adjusted Model 3 (OR = 1.002, *p* = 0.168), (Table 3).

**Table 3:** Multivariate logistic regression analyses for the associations between nutrition index, electrolyte index, inflammation index and insulin resistance index and PSI in different models.

Index	Variables	Model 1		Model 2		Model 3	
		OR (95%CI)	*p*	OR (95%CI)	*p*	OR (95%CI)	*p*
Nutrition index	GNRI	0.941 (0.932–0.950)	<0.001	0.960 (0.950–0.969)	<0.001	0.964 (0.954–0.974)	<0.001
	PNI	0.916 (0.905–0.926)	<0.001	0.938 (0.927–0.950)	<0.001	0.943 (0.931–0.955)	<0.001
Electrolyte index	Serum sodium	0.956 (0.939–0.974)	<0.001	0.959 (0.941–0.977)	<0.001	0.967 (0.948–0.986)	0.001
	Serum potassium	1.404 (1.213–1.626)	<0.001	1.272 (1.094–1.480)	0.002	1.122 (0.959–1.313)	0.151
Inflammation index	NLR	1.122 (1.103–1.141)	<0.001	1.111 (1.092–1.131)	<0.001	1.108 (1.089–1.128)	<0.001
	PLR	1.004 (1.003,1.004)	<0.001	1.003 (1.003, 1.004)	<0.001	1.003 (1.003, 1.004)	<0.001
Insulin resistance index	TyG	1.705 (1.561–1.863)	<0.001	1.932 (1.758–2.125)	<0.001	2.057 (1.848–2.293)	<0.001
	TyG-BMI	1.000 (0.998–1.002)	0.911	1.004 (1.002–1.006)	<0.001	1.002 (0.999–1.004)	0.168

Model 1 was adjusted for none; Model 2 adjusts for age and sex; Model 3 was adjusted for age, sex, past medical history (stroke, hypertension, diabetes, atrial fibrillation, Coronary heart disease and renal insufficiency).

### 3.4 The nonlinear relationship between the nutrition, electrolyte, inflammation, insulin resistance indices and PSI risk

**Figure 3:**
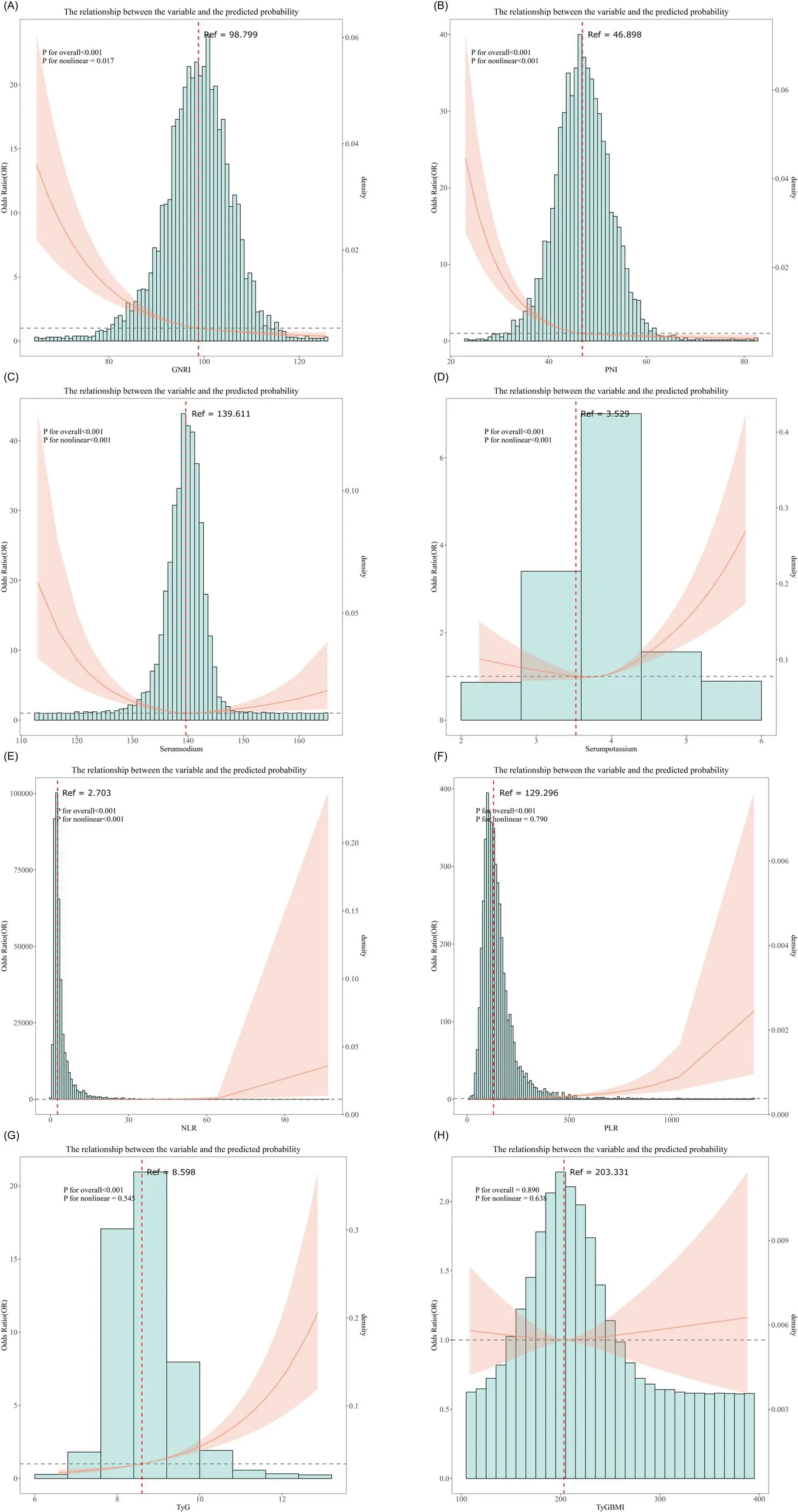
Restricted cubic spline curves depicting the associations of the nutritional index, electrolyte index, inflammation index, and insulin resistance index with post-stroke Infection.

To investigate the persistent association between nutritional indices, electrolyte indices, inflammatory indices, and insulin resistance indices with PSI, RCS analyses were performed with PSI as the dependent variable, adjusting for age, sex, and past medical history. Knots were placed at the 10th, 50th, and 90th percentiles of each predictor’s distribution (3 knots in total), as recommended by Harrell. The reference value was set at the median (50th percentile) of each index. The overall and nonlinear associations were tested using Wald tests. For all indices showing a significant overall association (P for overall < 0.05), the shape of the dose–response curve was further examined. The results revealed that GNRI, PNI, serum sodium, serum potassium, and NLR exhibited statistically significant nonlinear relationships with PSI (all P for nonlinear < 0.05), whereas TyG showed a linear relationship with PSI risk. For GNRI, the RCS curve crossed the reference line (OR = 1) at approximately 98.799. Above this value, the odds of PSI decreased as GNRI increased; below it, the odds tended to increase. For PNI, the curve crossed the reference line at approximately 46.898. Below this value, PSI risk showed an inverse trend with PNI; above it, the curve flattened, suggesting no further substantial reduction in risk with additional increases in PNI. For serum sodium, a U-shaped relationship with PSI was observed (P for nonlinear < 0.001). Below the inflection point (139.611 mmol/L), the risk of PSI increased with decreasing serum sodium; above the inflection point, the risk increased with rising serum sodium, confirming a U-shaped association. For serum potassium, a similar U-shaped pattern was detected (P for nonlinear < 0.001), with the reference crossing point at approximately 3.529 mmol/L. Above this value, higher potassium was associated with increased PSI risk; below it, the risk decreased as potassium increased. For NLR, the curve crossed the reference line at approximately 2.703. Below this value, increasing NLR did not significantly elevate PSI risk; above it, PSI risk rose sharply. A similar pattern was observed for PLR, with the reference crossing at approximately 129.296. For TyG, only a linear association was detected (P for nonlinear > 0.05), with higher TyG values associated with progressively increased PSI risk (Figure 3).

### 3.5 Comparison of the predictive value of nutrition, electrolyte, inflammation, insulin resistance indices

To comprehensively evaluate the predictive performance of various indices for post-stroke infection (PSI), we conducted a 5-fold cross-validated logistic regression analysis on eight candidate biomarkers categorized into four groups: nutrition indices, inflammation indices, electrolyte indices, and insulin resistance indices. Among the nutrition indices, the GNRI exhibited the highest predictive value with an AUC of 0.632 (95% CI: 0.615–0.649), accompanied by a sensitivity of 0.577, specificity of 0.634, and Youden index of 0.211. The PNI showed slightly lower but comparable performance, with an AUC of 0.614 (95% CI: 0.596–0.632), sensitivity of 0.571, specificity of 0.611, and Youden index of 0.203. For inflammation indices, the NLR demonstrated superior predictive ability compared to the PLR. NLR achieved an AUC of 0.617 (95% CI: 0.598–0.634), with a sensitivity of 0.477, specificity of 0.723, and Youden index of 0.200. In contrast, PLR showed relatively lower performance, with an AUC of 0.577 (95% CI: 0.560–0.596), sensitivity of 0.456, specificity of 0.689, and Youden index of 0.145. Among electrolyte indices, serum sodium exhibited better predictive performance than serum potassium. Serum sodium achieved an AUC of 0.548 (95% CI: 0.530–0.565), with a sensitivity of 0.450, specificity of 0.661, and Youden index of 0.111. Serum potassium showed the lowest predictive value among all electrolyte indices, with an AUC of 0.537 (95% CI: 0.518–0.555), sensitivity of 0.367, specificity of 0.725, and Youden index of 0.092. Regarding insulin resistance indices, the Triglyceride-glucose (TyG) index demonstrated moderate predictive ability with an AUC of 0.601 (95% CI: 0.583–0.618), sensitivity of 0.578, specificity of 0.597, and Youden index of 0.175. However, the TyG-BMI combination index showed the lowest overall predictive performance among all evaluated indices, with an AUC of 0.482 (95% CI: 0.465–0.522), sensitivity of 0.631, specificity of 0.394, and Youden index of 0.025, (Table 4).

**Table 4:** ROC curve analysis of nutrition index, electrolyte index, inflammation index and insulin resistance index.

Index		AUC (95%CI)	Sensitivity	Specificity	Youden index
Nutrition index	GNRI	0.632 (0.615–0.649)	0.577	0.634	0.211
	PNI	0.614 (0.596–0.632)	0.571	0.611	0.203
Inflammation index	NLR	0.617 (0.598–0.634)	0.477	0.723	0.200
	PLR	0.577 (0.560–0.596)	0.456	0.689	0.145
Electrolyte index	Serum sodium	0.548 (0.530–0.565)	0.450	0.661	0.111
	Serum potassium	0.537 (0.518–0.555)	0.367	0.725	0.092
Insulin resistance index	TyG	0.601 (0.583–0.618)	0.578	0.597	0.175
	TyG-BMI	0.482 (0.465–0.522)	0.631	0.394	0.025

### 3.6 Establish a predictive model based on the integration of nutritional index, electrolyte index, inflammation index, and insulin resistance index

**Figure 4:**
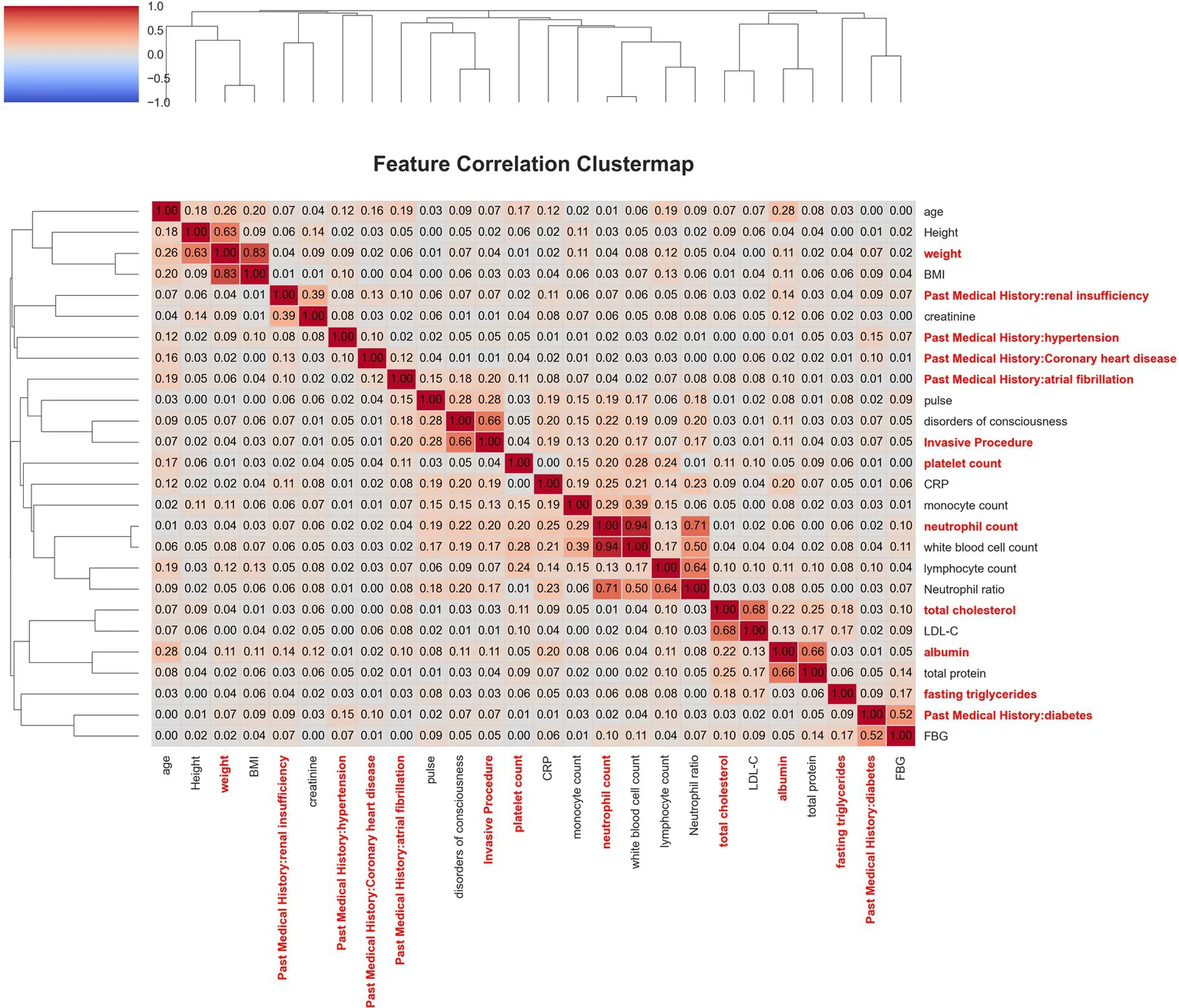
Hierarchical clustering heatmap identifying key features for post-stroke infection risk prediction.

In this study, hierarchical clustering was employed to identify clinical features with statistically significant differences (*p* < 0.05) in univariate analysis within the training dataset. Hierarchical clustering utilizes similarity measures between features (correlation coefficients) and progressively merges features into distinct clusters through bottom-up agglomerative methods. 12 features were ultimately selected. Specifically, these include weight, invasive procedure, past medical history (diabetes, hypertension, atrial fibrillation, renal insufficiency, Coronary heart disease),fasting triglycerides, total cholesterol, neutrophil count, platelet count, and albumin (Figure 4).

To systematically evaluate the combined predictive value of clinical features and biological indicators, we first selected the single indicator with the best predictive performance from each of the four major indicator systems by comparing ROC curve AUC values: GNRI for nutritional index, serum sodium for electrolyte index, NLR for inflammatory index, and TyG for insulin resistance index. Subsequently, these four selected indices were fused with 12 variables screened from clinical features using hierarchical clustering method, with raw indicators directly related to index calculation being eliminated during the fusion process. Based on this feature set, five machine learning algorithms(XGBoost, Lightgbm, RF, ET, CatBoost)were used to construct prediction models. The models were comprehensively evaluated from multiple dimensions including discriminative ability, clinical value, prediction consistency, and feature contribution using ROC curves, decision curve analysis (DCA), calibration curves, Delong’s test, and SHAP plots.

**Figure 5:**
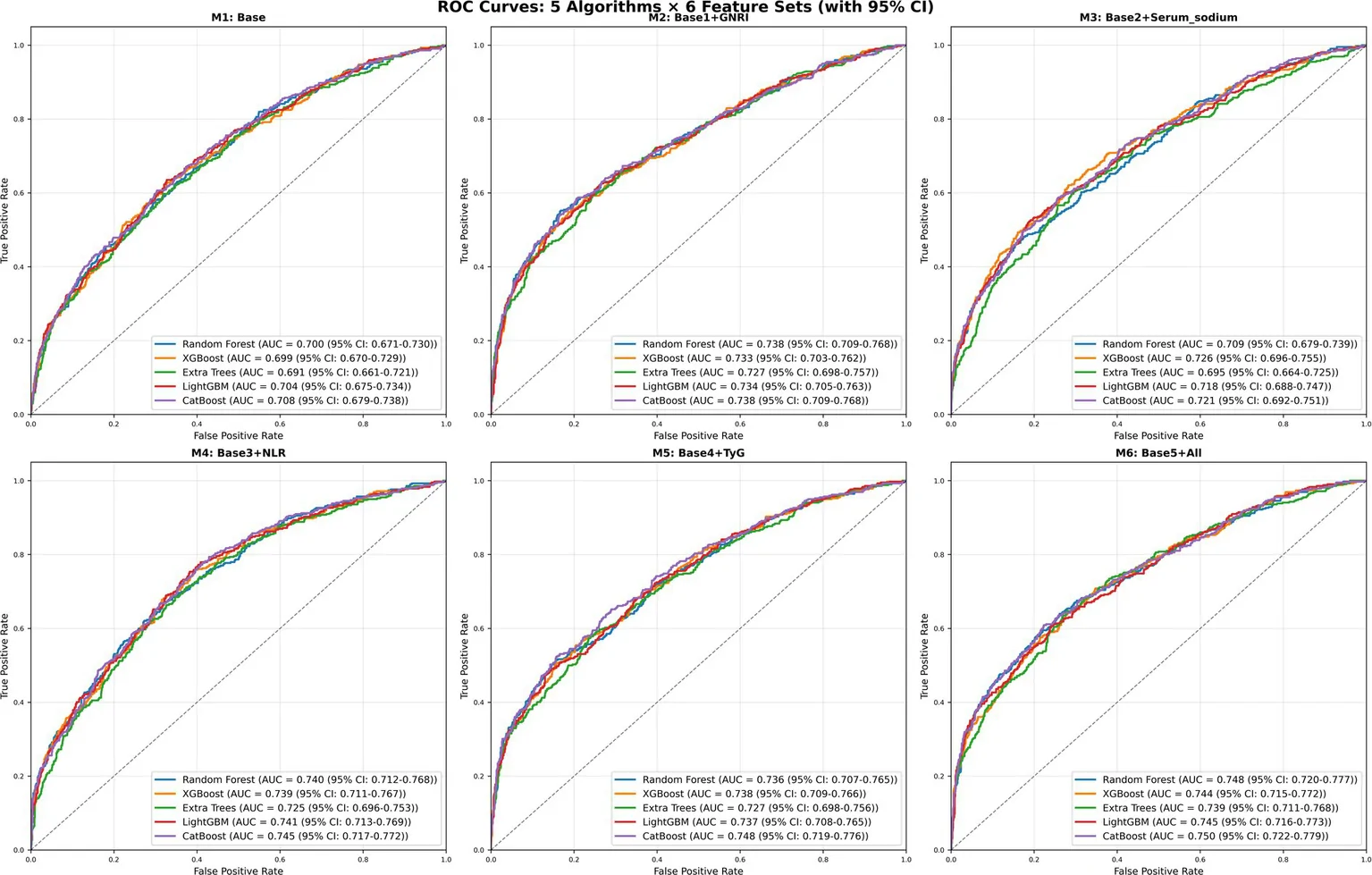
ROC curves of five algorithms across M1-M6 datasets for post-stroke infection prediction.

On the test set, the CatBoost model incorporating all four biomarkers (M6) achieved the highest discriminative performance (AUC = 0.750, 95% CI: 0.722–0.779; Youden index = 0.386). Among single-biomarker additions, CatBoost with NLR yielded the highest sensitivity (0.794), while LightGBM with GNRI achieved the highest specificity (0.856), indicating that the optimal algorithm–biomarker pairing depends on the clinical priority (screening sensitivity vs. diagnostic specificity) (Table 5; Figure 5).

**Table 5:** Comparison of prediction model performance across different data sets.

Algorithm	Feature set	AUC (95% CI)	Sensitivity	Specificity	Youden index	Optimal threshold	Brier score
CatBoost	M1: Base	0.708 (0.679–0.738)	0.607	0.696	0.303	0.485	0.2056
	M2: Base1 + GNRI	0.738 (0.709–0.768)	0.585	0.793	0.378	0.504	0.1927
	M3: Base2 + Serum_sodium	0.721 (0.692–0.751)	0.588	0.742	0.33	0.487	0.2015
	M4: Base3 + NLR	0.745 (0.717–0.772)	0.794	0.571	0.365	0.403	0.1963
	M5: Base4 + TyG	0.748 (0.719–0.776)	0.524	0.843	0.366	0.54	0.1893
	M6: Base5 + All	0.750 (0.722–0.779)	0.609	0.777	0.386	0.485	0.1858
Extra Trees	M1: Base	0.691 (0.661–0.721)	0.597	0.681	0.278	0.491	0.2343
	M2: Base1 + GNRI	0.727 (0.698–0.757)	0.595	0.754	0.349	0.441	0.2111
	M3: Base2 + Serum_sodium	0.695 (0.664–0.725)	0.566	0.744	0.31	0.442	0.2237
	M4: Base3 + NLR	0.725 (0.696–0.753)	0.685	0.653	0.338	0.455	0.2267
	M5: Base4 + TyG	0.727 (0.698–0.756)	0.573	0.765	0.338	0.437	0.2102
	M6: Base5 + All	0.739 (0.711–0.768)	0.647	0.721	0.368	0.436	0.2101
LightGBM	M1: Base	0.704 (0.675–0.734)	0.635	0.673	0.308	0.469	0.2055
	M2: Base1 + GNRI	0.734 (0.705–0.763)	0.507	0.856	0.363	0.561	0.1947
	M3: Base2 + Serum_sodium	0.718 (0.688–0.747)	0.524	0.811	0.335	0.522	0.2014
	M4: Base3 + NLR	0.741 (0.713–0.769)	0.754	0.618	0.371	0.416	0.1955
	M5: Base4 + TyG	0.737 (0.708–0.765)	0.491	0.855	0.345	0.551	0.1914
	M6: Base5 + All	0.745 (0.716–0.773)	0.607	0.759	0.366	0.475	0.1862
Random Forest	M1: Base	0.700 (0.671–0.730)	0.581	0.7	0.281	0.485	0.2119
	M2: Base1 + GNRI	0.738 (0.709–0.768)	0.552	0.832	0.384	0.515	0.2011
	M3: Base2 + Serum_sodium	0.709 (0.679–0.739)	0.464	0.843	0.308	0.515	0.211
	M4: Base3 + NLR	0.740 (0.712–0.768)	0.671	0.678	0.349	0.459	0.2051
	M5: Base4 + TyG	0.736 (0.707–0.765)	0.514	0.843	0.358	0.515	0.2006
	M6: Base5 + All	0.748 (0.720–0.777)	0.64	0.739	0.379	0.465	0.1968
XGBoost	M1: Base	0.699 (0.670–0.729)	0.628	0.672	0.3	0.457	0.2047
	M2: Base1 + GNRI	0.733 (0.703–0.762)	0.547	0.814	0.361	0.532	0.194
	M3: Base2 + Serum_sodium	0.726 (0.696–0.755)	0.621	0.717	0.338	0.468	0.1986
	M4: Base3 + NLR	0.739 (0.711–0.767)	0.758	0.605	0.364	0.406	0.1942
	M5: Base4 + TyG	0.738 (0.709–0.766)	0.555	0.796	0.351	0.501	0.1898
	M6: Base5 + All	0.744 (0.715–0.772)	0.637	0.724	0.361	0.45	0.1863

M1: Base: the 12 features obtained from hierarchical clustering. M2: Base1 + GNRI: after excluding albumin and weight from the 12 features derived via hierarchical clustering, the remaining 10 features were integrated with the GNRI, yielding a final set of 11 features. M3: Base2 + Serum_sodium: the 12 features derived from hierarchical clustering were integrated with serum sodium, yielding a total of 13 features. M4: Base3 + NLR: after excluding neutrophil count from the 12 features derived via hierarchical clustering, the remaining 11 features were integrated with the NLR, yielding a final set of 12 features. M5: Base4 + TyG: after excluding fasting triglyceride from the 12 features derived via hierarchical clustering, the remaining 11 features were integrated with the TyG, yielding a final set of 12 features. M6: Base5 + All: after excluding albumin, weight, neutrophil count and fasting triglyceride from the 12 features derived via hierarchical clustering, the remaining 8 features were integrated with the GNRI, Serum sodium, NLR and TyG, yielding a final set of 12 features.

DCA demonstrated that the CatBoost model (M6) yielded superior net benefit over all comparator models across threshold probabilities of 0.04–0.33 (net benefit range: 0.091–0.256). At thresholds beyond 0.47, net benefit declined to negligible levels (<0.07), suggesting limited additional clinical utility in very high-risk scenarios (Figure 5).

**Figure 6:**
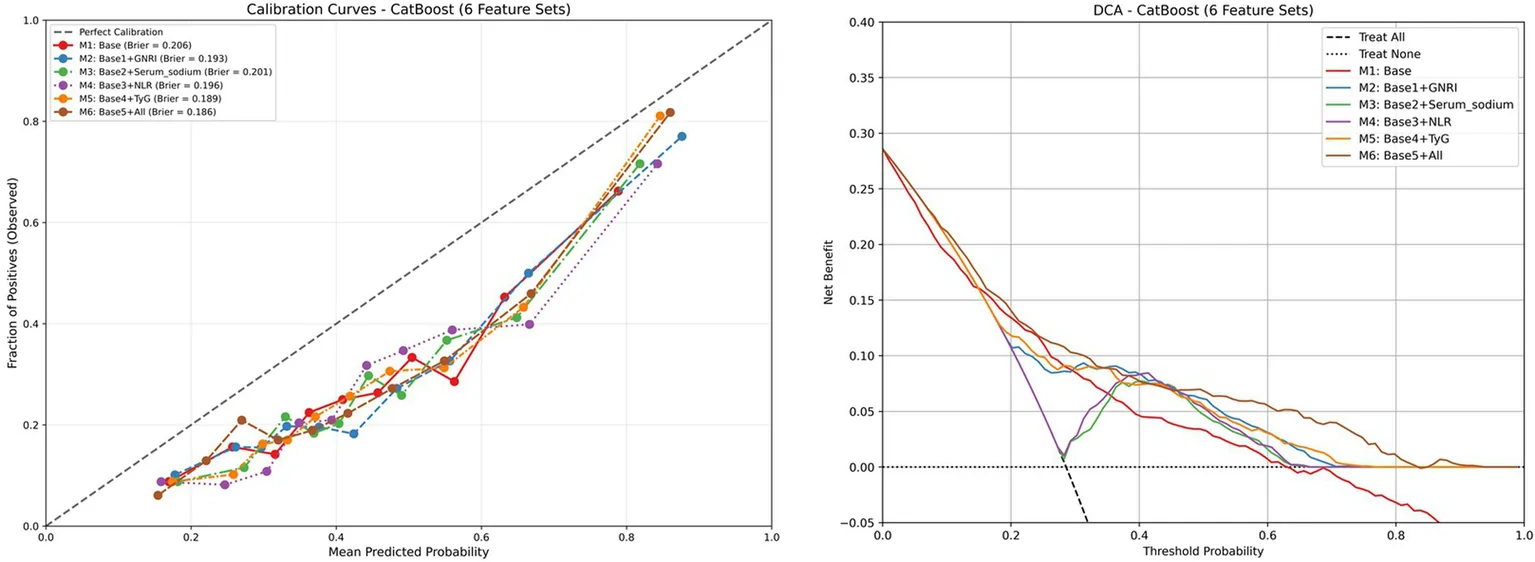
Calibration curves and decision curve analyses of the optimal CatBoost model across M1-M6 datasets.

Calibration assessment confirmed that M6 achieved the lowest Brier score (0.186) and the closest approximation to the identity line, indicating strong agreement between predicted and observed probabilities (Figure 6).

**Figure 7:**
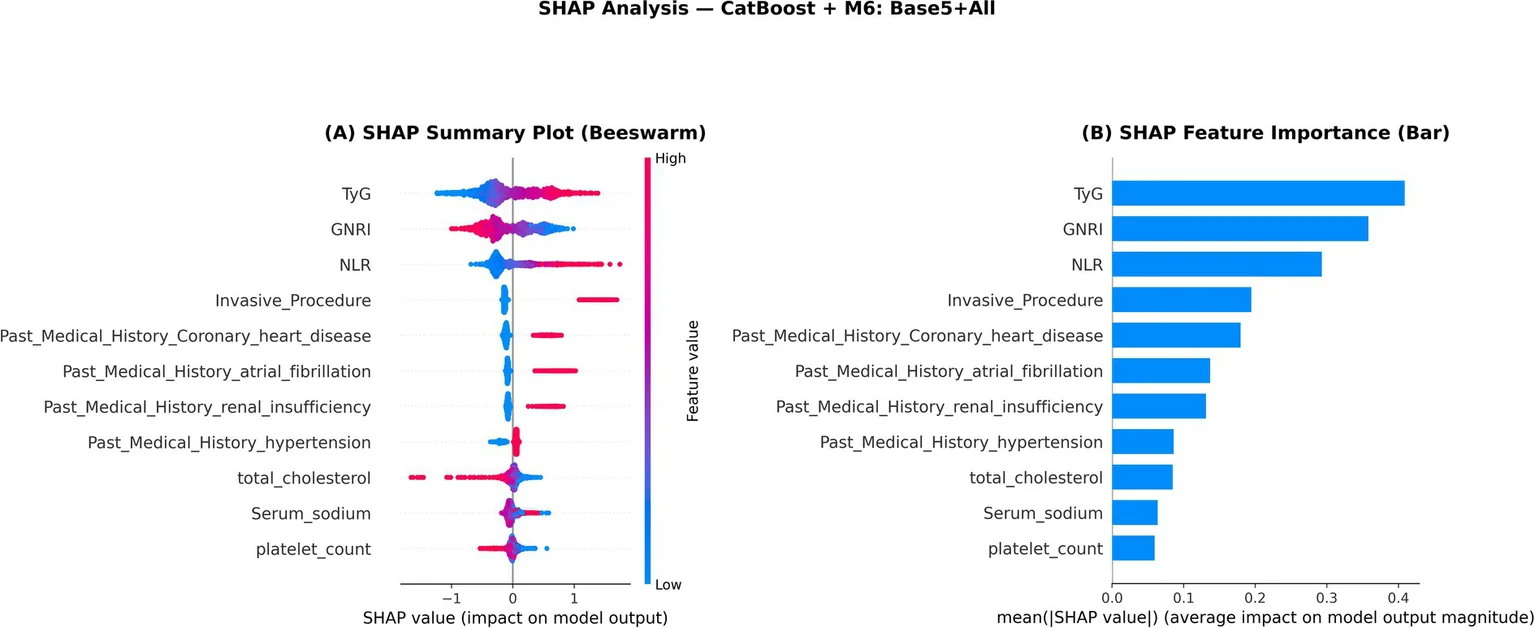
SHAP analysis of top contributing features in CatBoost model (m6: base5+all dataset) for post-stroke infection prediction.

SHAP analysis identified the top contributing features in the full model: TyG (mean |SHAP| = 0.409), GNRI (0.358), NLR (0.293), invasive procedure (0.194), coronary heart disease (0.179), and atrial fibrillation (0.137) (Figure 7). Higher TyG and NLR values, and lower GNRI, were associated with elevated predicted PSI risk, consistent with their established roles in metabolic dysregulation, systemic inflammation, and immunodeficiency, respectively.

### 3.7 Webpage deployment tool

**Figure 8:**
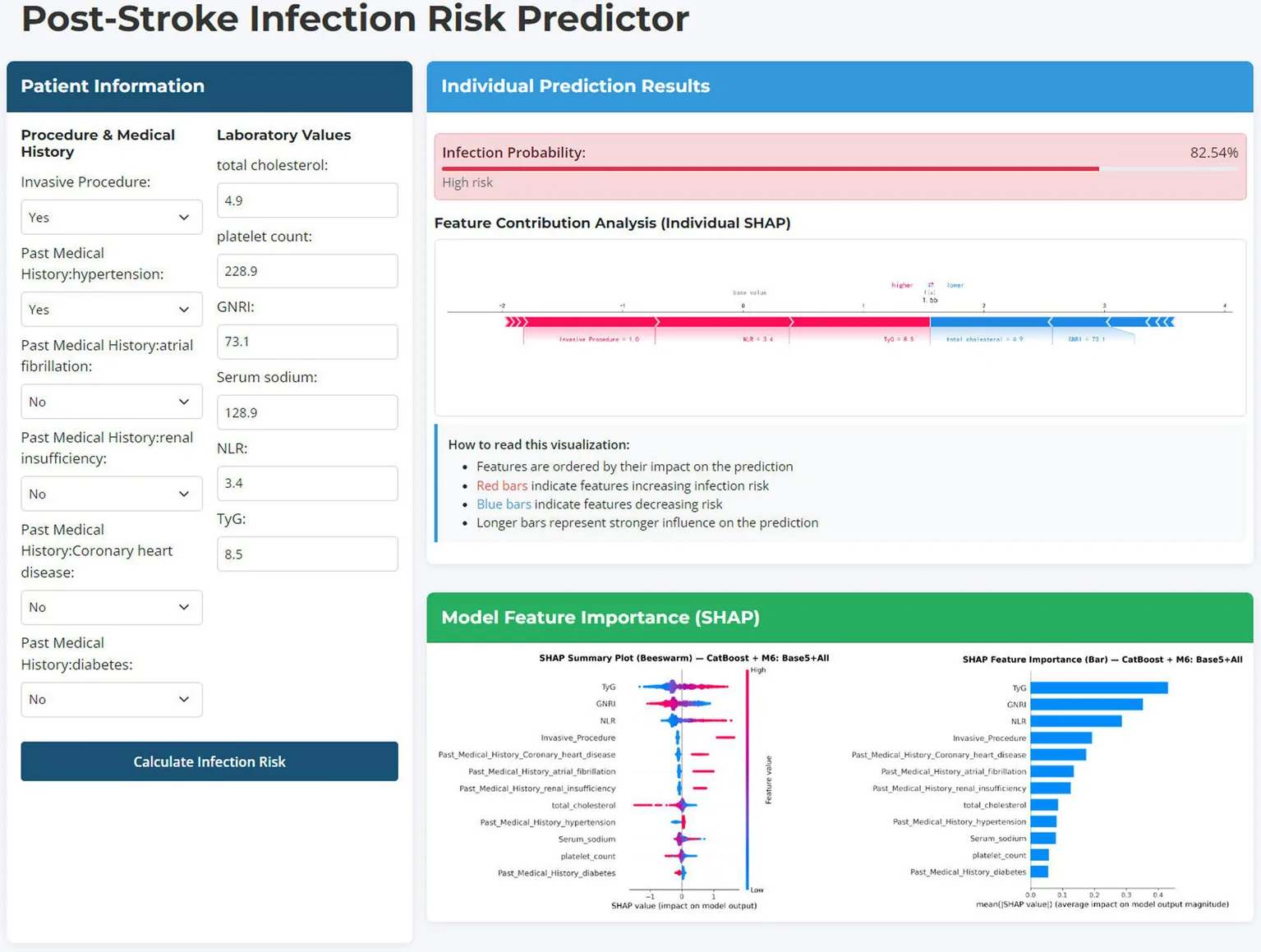
Web-based post-stroke infection risk prediction platform integrated with CatBoost model.

Based on the M6: Base5 + All dataset, the CatBoost model exhibited the best predictive performance. Accordingly, we developed a web-based prediction platform integrated with the CatBoost model (Figure 8). By processing user-input predictors, calculating the risk of Post-Stroke Infection, and presenting the results in a clear and intuitive manner on web-based plots, this application provides personalized risk assessments.

## 4 Discussion

PSI is a significant determinant of adverse outcomes in stroke patients, with incidence rates reported to vary widely between 5 and 65% (17). In addition to directly increasing disability and mortality rates, thereby adversely affecting patient prognosis, PSI extends hospital stays, impedes rehabilitation progress, and imposes considerable economic burdens on families and society (18, 19). Given the substantial clinical and socioeconomic ramifications of PSI, there is an urgent need for reliable tools to identify high-risk patients early and implement targeted interventions. However, existing research on PSI risk prediction has predominantly concentrated on individual biological indicators, failing to account for the multifactorial nature of this complication.

To address this critical gap, we systematically evaluated four interconnected biological domains—nutritional status, electrolyte balance, systemic inflammation, and insulin resistance—and their combined predictive utility for PSI in patients with AIS. Our findings demonstrate that GNRI, serum sodium, NLR, and TyG are independent predictors of PSI, and that their integration with clinical features in a machine learning framework yields clinically meaningful discriminative performance.

Malnutrition is a well-established risk factor for post-stroke complications. Our analysis confirmed that lower GNRI and PNI were independently associated with increased PSI risk, consistent with evidence that protein-energy malnutrition impairs lymphocyte proliferation, complement activity, and mucosal barrier integrity (20–23). Importantly, RCS analysis identified threshold effects (GNRI ≤ 98.8, PNI ≤ 46.9), below which PSI risk escalated sharply, while above these inflection points the risk plateaued. These findings suggest that nutritional interventions may confer the greatest infection-prevention benefit among patients with moderate-to-severe malnutrition (7, 24), whereas indiscriminate supplementation in well-nourished individuals may offer limited incremental protection (18, 25). Compared with prior studies that used single markers such as albumin or lymphocyte count alone, the composite GNRI and PNI provide a more integrative assessment of nutritional–immune status.

Serum sodium and potassium levels both exhibit U-shaped associations with PSI risk. Serum sodium shows an inflection point at 139.611 mmol/L, with risk decreasing as sodium rises below this threshold and increasing above it. Serum potassium has an inflection point at 3.529 mmol/L, where both lower and higher values confer elevated risk. These findings confirm a direct association between electrolyte disturbances (hyponatremia/hypernatremia, hypokalemia/hyperkalemia) and impaired immune function (26). Current research demonstrates that sodium plays a pivotal role in immune responses and tissue homeostasis. Sodium intake can directly modulate the activation status of the immune system by influencing T-helper cell subsets and innate immune cells (8). Specifically, One pivotal study identifies hyponatremia as an independent inducer of neutrophil extracellular traps (NETs) release, a process that is closely linked to neutrophil function and chemotaxis (27). Emerging evidence indicates that high salt intake alters gut microbiota composition, thereby indirectly modulating immune cell activity (28). These findings collectively suggest that sodium intake and metabolism may influence PSI risk through immune response regulation (29). Notably, serum potassium showed significance only in unadjusted models during multivariate regression analysis, implying its potential confounding by underlying conditions (e.g., renal dysfunction) or therapeutic interventions (e.g., diuretics). Clinical decision-making thus requires comprehensive evaluation of patient-specific contexts.

Inflammatory responses have been confirmed as core drivers for predicting PSI. NLR and PLR, as convenient indicators reflecting systemic inflammatory status, showed significant positive correlations with PSI risk across all adjusted models. RCS analysis identified clinical thresholds for NLR and PLR (2.703 and 129.296, respectively). Above these thresholds, risk rose markedly. When NLR or PLR remains below the threshold, the body may maintain immune balance through compensatory mechanisms. Once the threshold is exceeded, excessive inflammatory responses exacerbate tissue damage and suppress immune function, thereby increasing infection risk. This perspective is supported by multiple studies. For instance, in a study of sepsis patients, researchers found elevated NLR and PLR levels significantly correlated with 28-day mortality, indicating these markers serve as important predictors of prognosis in sepsis patients (30). Another study examined the prognostic value of NLR and PLR in head and neck cancer patients, revealing that high NLR and PLR levels were associated with poorer survival rates (31). This further supports the role of NLR and PLR as markers of inflammatory and immune dysregulation, particularly when these ratios exceed specific thresholds, potentially indicating higher disease risk and poorer prognosis. Furthermore, SHAP analysis revealed that NLR is one of the most significant contributors in the combined model, further validating its clinical value as an inflammatory activity marker.

TyG is linearly positively correlated with PSI risk, and the effect is enhanced with the addition of adjusted variables (Model 3: OR = 2.057), suggesting that insulin resistance is a strong independent predictor of PSI. Insulin resistance promotes infections through multiple mechanisms. Notably, hyperglycemic conditions enhance NADPH oxidase activity in endothelial microparticles, which exacerbates endothelial inflammation and induces functional impairment (32); On the other hand, chronic inflammation associated with insulin resistance (such as elevated TNF-*α* and IL-6) can exacerbate vascular endothelial injury and increase the risk of bacterial translocation (33). This study did not find an association between TyG-BMI and PSI, possibly due to BMI’s insufficient ability to distinguish body fat distribution. In contrast, the TyG index relies solely on fasting blood glucose and triglycerides, providing a more direct reflection of IR severity and thus demonstrating superior predictive performance.

The integration of four biomarkers with clinical features in a CatBoost model (M6) achieved an AUC of 0.750, outperforming all single-index models, and demonstrated satisfactory calibration (Brier score: 0.186). DCA confirmed clinical net benefit across threshold probabilities of 0.04–0.33, supporting its applicability for early risk screening. SHAP analysis identified TyG, GNRI, NLR, invasive procedures, coronary heart disease, and atrial fibrillation as the principal contributors, reflecting the interplay of metabolic, nutritional, inflammatory, and procedural risk factors. The web-based calculator (Figure 8) enables bedside risk assessment, allowing clinicians to identify high-risk patients and consider early interventions—such as intensified nutritional support, glycemic optimization, or prophylactic measures—before clinical signs of infection emerge.

In conclusion, malnutrition (GNRI ≤ 98.8, PNI ≤ 46.9), electrolyte disturbances (sodium < 139.6 or > 139.6 mmol/L), systemic inflammation (NLR > 2.7, PLR > 129.3), and insulin resistance (elevated TyG index) are independent predictors of PSI in AIS patients. A machine learning model integrating these indices with clinical features provides robust discriminative performance and clinical net benefit, offering a practical tool for early risk stratification.

This study has the following limitations:. First, as a single-center retrospective study, key variables—including NIHSS score, dysphagia status, and consciousness level—were not systematically recorded, precluding their inclusion as covariates. Residual confounding may therefore persist, and our findings should be interpreted as associations rather than causal relationships. Second, the absence of serial biomarker measurements limits our ability to assess temporal relationships with infection onset. Third, the lack of an external validation cohort necessitates further prospective, multicenter studies to confirm generalizability. Future work should also investigate whether combined interventions can reduce PSI incidence in high-risk patients identified by this model.

## Data Availability

The original contributions presented in the study are included in the article/supplementary material, further inquiries can be directed to the corresponding author.
